# Role of sex hormones in modulating myocardial perfusion and coronary flow reserve

**DOI:** 10.1007/s00259-022-05675-2

**Published:** 2022-01-13

**Authors:** Achi Haider, Susan Bengs, Angela Portmann, Alexia Rossi, Hazem Ahmed, Dominik Etter, Geoffrey I. Warnock, Nidaa Mikail, Muriel Grämer, Alexander Meisel, Livio Gisler, Caitlin Jie, Claudia Keller, Sebastian Kozerke, Bruno Weber, Roger Schibli, Linjing Mu, Philipp A. Kaufmann, Vera Regitz-Zagrosek, Simon M. Ametamey, Catherine Gebhard

**Affiliations:** 1 Department of Nuclear Medicine, University Hospital Zurich, Raemistrasse 100, CH-8091 Zurich, Switzerland; 2 Center for Molecular Cardiology, University of Zurich, CH-8952 Schlieren, Switzerland; 3 Institute of Pharmaceutical Sciences, ETH Zurich, CH-8093 Zurich, Switzerland; 4 Institute for Biomedical Engineering, University and ETH Zurich, CH-8092 Zurich, Switzerland; 5 Institute of Pharmacology and Toxicology, University of Zurich, CH-8057 Zurich, Switzerland; 6 Institute for Gender in Medicine, Charité Universitaetsmedizin Berlin, D-10115 Berlin, Germany; 7 University Hospital Zurich, CH-8091 Zurich, Switzerland

**Keywords:** Rest/stress myocardial perfusion imaging (MPI), Positron emission tomography (PET), [^18^F]flurpiridaz, Sex hormones, Sex differences, Coronary flow reserve (CFR)

## Abstract

**Background:**

A growing body of evidence highlights sex differences in the diagnostic accuracy of cardiovascular imaging modalities. Nonetheless, the role of sex hormones in modulating myocardial perfusion and coronary flow reserve (CFR) is currently unclear. The aim of our study was to assess the impact of female and male sex hormones on myocardial perfusion and CFR.

**Methods:**

Rest and stress myocardial perfusion imaging (MPI) was conducted by small animal positron emission tomography (PET) with [^18^F]flurpiridaz in a total of 56 mice (7–8 months old) including gonadectomized (Gx) and sham-operated males and females, respectively. Myocardial [^18^F]flurpiridaz uptake (% injected dose per mL, % ID/mL) was used as a surrogate for myocardial perfusion at rest and following intravenous regadenoson injection, as previously reported. Apparent coronary flow reserve (CFR_App_) was calculated as the ratio of stress and rest myocardial perfusion. Left ventricular (LV) morphology and function were assessed by cardiac magnetic resonance (CMR) imaging.

**Results:**

Orchiectomy resulted in a significant decrease of resting myocardial perfusion (Gx vs. sham, 19.4 ± 1.0 vs. 22.2 ± 0.7 % ID/mL, p = 0.034), while myocardial perfusion at stress remained unchanged (Gx vs. sham, 27.5 ± 1.2 vs. 27.3 ± 1.2 % ID/mL, p = 0.896). Accordingly, CFR_App_ was substantially higher in orchiectomized males (Gx vs. sham, 1.43 ± 0.04 vs. 1.23 ± 0.05, p = 0.004), and low serum testosterone levels were linked to a blunted resting myocardial perfusion (*r* = 0.438, p = 0.020) as well as an enhanced CFR_App_ (*r* = −0.500, p = 0.007). In contrast, oophorectomy did not affect myocardial perfusion in females. Of note, orchiectomized males showed a reduced LV mass, stroke volume, and left ventricular ejection fraction (LVEF) on CMR, while no such effects were observed in oophorectomized females.

**Conclusion:**

Our experimental data in mice indicate that sex differences in myocardial perfusion are primarily driven by testosterone. Given the diagnostic importance of PET-MPI in clinical routine, further studies are warranted to determine whether testosterone levels affect the interpretation of myocardial perfusion findings in patients.

**Supplementary Information:**

The online version contains supplementary material available at 10.1007/s00259-022-05675-2.

## Introduction

Positron emission tomography myocardial perfusion imaging (PET-MPI) is the most commonly used tool for the absolute quantification of myocardial blood flow (MBF). As such, impaired coronary flow reserve (CFR) derived from the ratio of stress and rest MBF by PET-MPI is the current non-invasive reference standard for the detection of coronary microvascular dysfunction [1]. Recently, [^18^F]flurpiridaz was introduced as a novel tracer for PET-MPI [2, 3]. [^18^F]flurpiridaz is a valid alternative to conventional PET-MPI tracers owing to its high myocardial extraction across a wide range of achievable flows, as well as its high spatial resolution [4]. The relatively long physical half-life of [^18^F]flurpiridaz obviates the need for an on-site cyclotron and allows treadmill stress testing. Of note, [^18^F]flurpiridaz PET proved to be particularly accurate for CFR measurements in patient populations where tissue attenuation correction is challenging — including women and obese patients — suggesting that challenges of tissue attenuation correction can be overcome by the relatively high spatial resolution of images obtained with [^18^F]flurpiridaz [3].

Molecular mechanisms affecting coronary microcirculation are still under investigation and care must be taken in the interpretation of decreased CFR values. Previous studies have reported sex differences in the prognostic value of PET-derived CFR [5] as well as higher resting MBF in asymptomatic women as compared to men [6]. Moreover, two previous studies indicate that coronary flow velocity reserve, which is an invasive measure of microvascular function, was lower in women with chest pain and unobstructed coronary arteries than in their male counterparts [7, 8]. Despite these documented sex differences, little is known about the effect of sex hormones on MBF and CFR. Therefore, the aim of our study was to investigate the impact of gonadectomy on myocardial perfusion and flow reserve in mice using [^18^F]flurpiridaz PET-MPI.

## Methods

### Gonadectomy

Female and male FVB/N mice were purchased from Janvier Labs (France). Prior to shipment, animals were randomized into subgroups that were subjected to either sham-surgery or gonadectomy (Gx) at the age of one month. All animal experiments were performed in accordance with the Swiss animal welfare laws and were approved by the Cantonal Veterinary Office of Zurich (Switzerland). Mice were housed under specific pathogen-free conditions in individually ventilated cages with continuous free access to water and food ad libitum.

### Rest/stress myocardial perfusion imaging

[^18^F]Flurpiridaz PET was conducted in a total of 56 animals (7–8 months) that were anesthetized with 1.3–1.9% isoflurane in oxygen-enriched air (1:1). Body temperature was monitored with a small rectal thermistor. [^18^F]Flurpiridaz was administered via tail-vein injection 60 s after scan start (0.6–8.2 MBq). Tracer distribution was recorded in dynamic PET acquisition mode over a time period of 41 min, before a mixture of regadenoson (0.1 μg/g), and a second dose of [^18^F]flurpiridaz was injected via a pre-installed intravenous catheter. In analogy to the rest scans, stress scans were acquired for 41 min. PET imaging was performed with a Super Argus PET/CT tomography (Sedecal, Spain), followed by a CT scan for anatomical information. PET data were reconstructed with a user-defined protocol at a voxel size of 0.3875 × 0.3875 × 0.775 mm^3^. PET data were processed with PMOD v.3.8 (PMOD Technologies, Switzerland). A volume of interest (VOI) delineating the myocardium was drawn manually by PMOD v.3.8 (PMOD Technologies, Switzerland). Average myocardial [^18^F]flurpiridaz uptake from 20 to 40 min post injection, calculated from % injected dose per mL of the myocardium (% ID/mL), was used as a surrogate for myocardial perfusion at rest and following intravenous regadenoson injection, as previously reported [9]. Apparent coronary flow reserve (CFR_App_) was calculated as the ratio between myocardial [^18^F]flurpiridaz uptake following vasodilator stress and at resting conditions. The term “apparent” is used because CFR_App_ values were calculated without an arterial input function [9].

### Cardiac magnetic resonance (CMR)

Mice were initially anesthetized with isoflurane (1.8–2.5%) in oxygen-enriched air. Body temperature was monitored with a small rectal thermistor probe (ERT model 1030 control/gating module, Small Animal Instruments Inc, USA) and maintained at 36–37°C using a warm-water circuit. All MRI experiments were performed on a Bruker BioSpec 70/30 USR magnetic resonance scanner (Bruker BioSpin MRI, Germany), equipped with a 1H receive-only 2×2 mouse cardiac surface array coil (Bruker BioSpin AG, Switzerland) and a Bruker console running ParaVision 6.0.1 (Bruker Cooperation, USA). For the two-chamber view of the LV long axis (LA), a cine-fast low angle shot (Cine-FLASH) sequence with simultaneous respiratory self-gating was performed using the following parameters: Field of view (FOV) = 25 mm × 25 mm, matrix dimension (MD) = 192 × 192, slice thickness (STH) = 0.8 mm, flip angle (FA) = 15, repetition time (ReT) = 8 ms, echo time (ET) = 2.4 ms, number of averages (NA) = 8 resulting in 12 frames. The longest distance of the LV cavity was determined between the mitral valves and the most apical point of the LV cavity. This length was used to plan subsequent coronal Cine-FLASH short axis (SA) scans. LVEF and stroke volume (SV) were calculated according to the modified Simpson rule at 1/3 (Am) and 2/3 (Ap) of the LV length [10].

### Heart rate reserve

Heart rate was monitored during CMR imaging by electrocardiogram (ECG) electrodes that were inserted subcutaneously into the paws of anesthetized animals (1.8–2.5% isoflurane in oxygen-enriched air) and recorded using SAII monitoring software (Small Animal Instruments Inc, USA). Heart rate was measured at rest and following pharmacological stress (5 min after regadenoson injection, 0.1 μg/g bodyweight). Heart rate reserve (HRR), calculated as (HR_Stress_–HR_Rest_)/HR_Rest_ * 100, was used to assess cardiac autonomic function, as previously reported in humans and mice [11, 12].

### Quantification of circulating sex hormones

Sex hormone levels were measured using commercially available ELISA kits, according to the manufacturer’s recommendation (Calbiotech, USA). The absorbance was determined at a wavelength of 450 nm and 570 nm for the pathlength correction using a Tecan infinite pro 200 reader (Tecan, Switzerland). A 4-parameter logistic regression algorithm was used to fit the standard curve. Mean sample hormone concentrations were calculated in a standard range of 0.1–18 ng/mL for testosterone. Progesterone levels were determined by LC-MS/MS, as previously reported [13].

### Statistical analysis

All statistical analyses were carried out with IBM SPSS Statistics 25 (IBM Corp., USA). Continuous variables are presented as mean ± standard error of the mean (SEM). Analysis of variance (ANOVA) and Student’s t-test were used for group comparisons of continuous variables. Strength and direction of associations were assessed by Spearman’s rank-order correlation. A p-value of ≤0.05 (two-tailed) was considered statistically significant.

## Results

### Orchiectomy is associated with reduced baseline myocardial perfusion and elevated CFR_App_

**Fig. 1:**
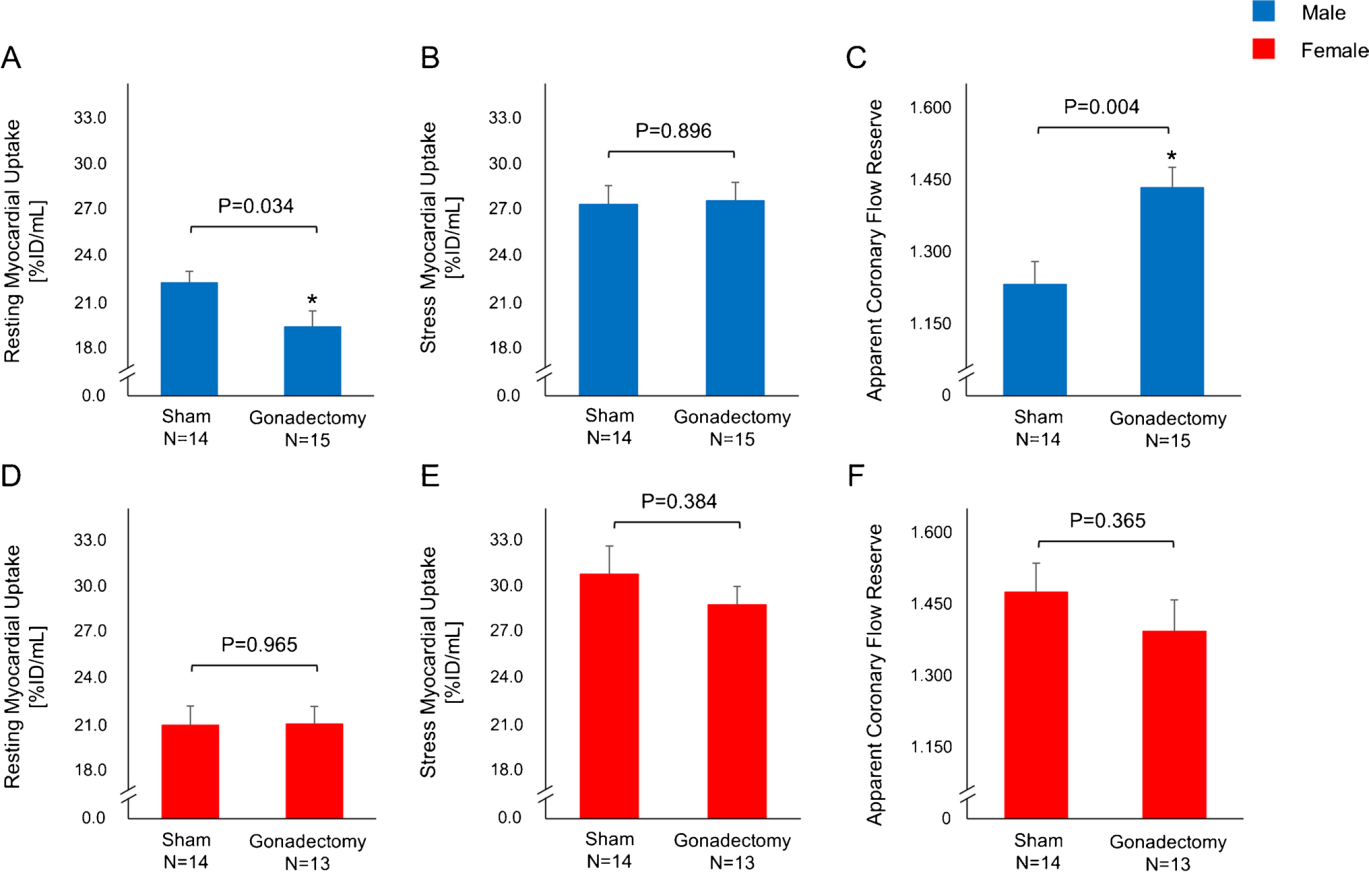
Rest/stress myocardial perfusion imaging by small animal positron emission tomography (PET). (**A**) Resting myocardial perfusion in males. (**B**) Stress myocardial perfusion in males. (**C**) Apparent coronary flow reserve (CFR_App_) in males. (**D**) Resting myocardial perfusion in females. (**E**) Stress myocardial perfusion in females. (**F**) Apparent coronary flow reserve (CFR_App_) in females

**Fig. 2:**
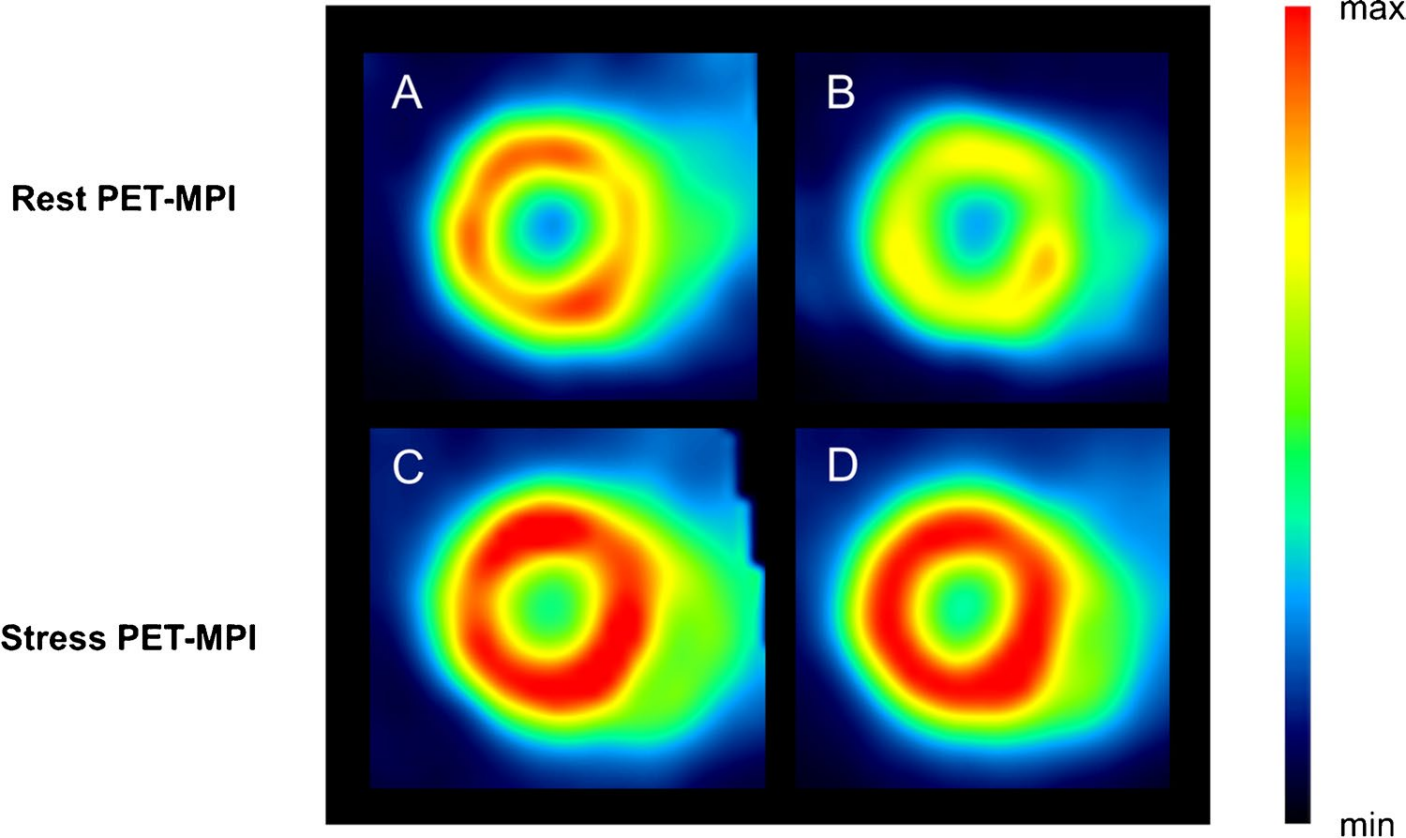
Representative positron emission tomography (PET) images of the mouse myocardium at rest and following pharmacological stress in sham and gonadectomized (Gx) males. (**A**) Sham, rest-MPI; (**B**) Gx, rest-MPI; (**C**) sham, stress-MPI; (**D**) Gx, stress-MPI

Gonadectomy was confirmed by assessment of serum sex hormone levels as well as by visual confirmation of the absence of testicular tissue in male mice and the absence of ovarian tissue in female mice at the time of organ harvesting. Testosterone levels were diminished in gonadectomized males, while progesterone serum concentrations were substantially reduced in gonadectomized females (Supplemental Fig. 1). Regadenoson administration resulted in fast coronary vasodilation, leading to an enhanced myocardial perfusion across all animal groups. At rest, sham males displayed a significantly higher myocardial perfusion (22.2 ± 0.7 % ID/mL), as compared to gonadectomized males (19.4 ± 1.0 % ID/mL, *p* = 0.034, Fig. 1A). In contrast, orchiectomy did not affect stress myocardial perfusion, as evidenced by the comparable myocardial [^18^F]flurpiridaz uptake in both groups (Gx vs. sham, 27.5 ± 1.2 vs. 27.3 ± 1.2 % ID/mL, *p* = 0.896, Fig. 1B). Consequently, CFR_App_ calculated as the ratio of stress to resting myocardial perfusion, was higher in gonadectomized males as compared to sham males (Gx vs. sham, 1.43 ± 0.04 vs. 1.23 ± 0.05, *p* = 0.004, Fig. 1C). Oophorectomy had no influence on baseline myocardial perfusion (Gx vs. sham, 21.1 ± 1.1 vs. 21.0 ± 1.2 % ID/mL, *p* = 0.965, Fig. 1D). Although there was a trend toward an attenuated myocardial perfusion at stress in gonadectomized females, this difference did not reach statistical significance (Gx vs. sham, 28.8 ± 1.2 vs. 30.8 ± 1.8 % ID/mL, *p* = 0.384, Fig. 1E). In concert, oophorectomy did not alter CFR_App_ in females (Gx vs. sham, 1.40 ± 0.07 vs. 1.48 ± 0.05, *p* = 0.365, Fig. 1F). A one-way ANOVA revealed that baseline CFR_App_ values were significantly higher in sham females than sham males (female vs. male, 1.48 ± 0.05 vs. 1.23 ± 0.05, *p* = 0.013), which was not the case for gonadectomized animals (female vs. male, 1.40 ± 0.07 vs. 1.43 ± 0.04, *p* = 0.951). Representative baseline and hyperemic PET-MPI images of the male myocardium are depicted in Fig. 2.

### Autonomic function is altered in gonadectomized males

**Fig. 3:**
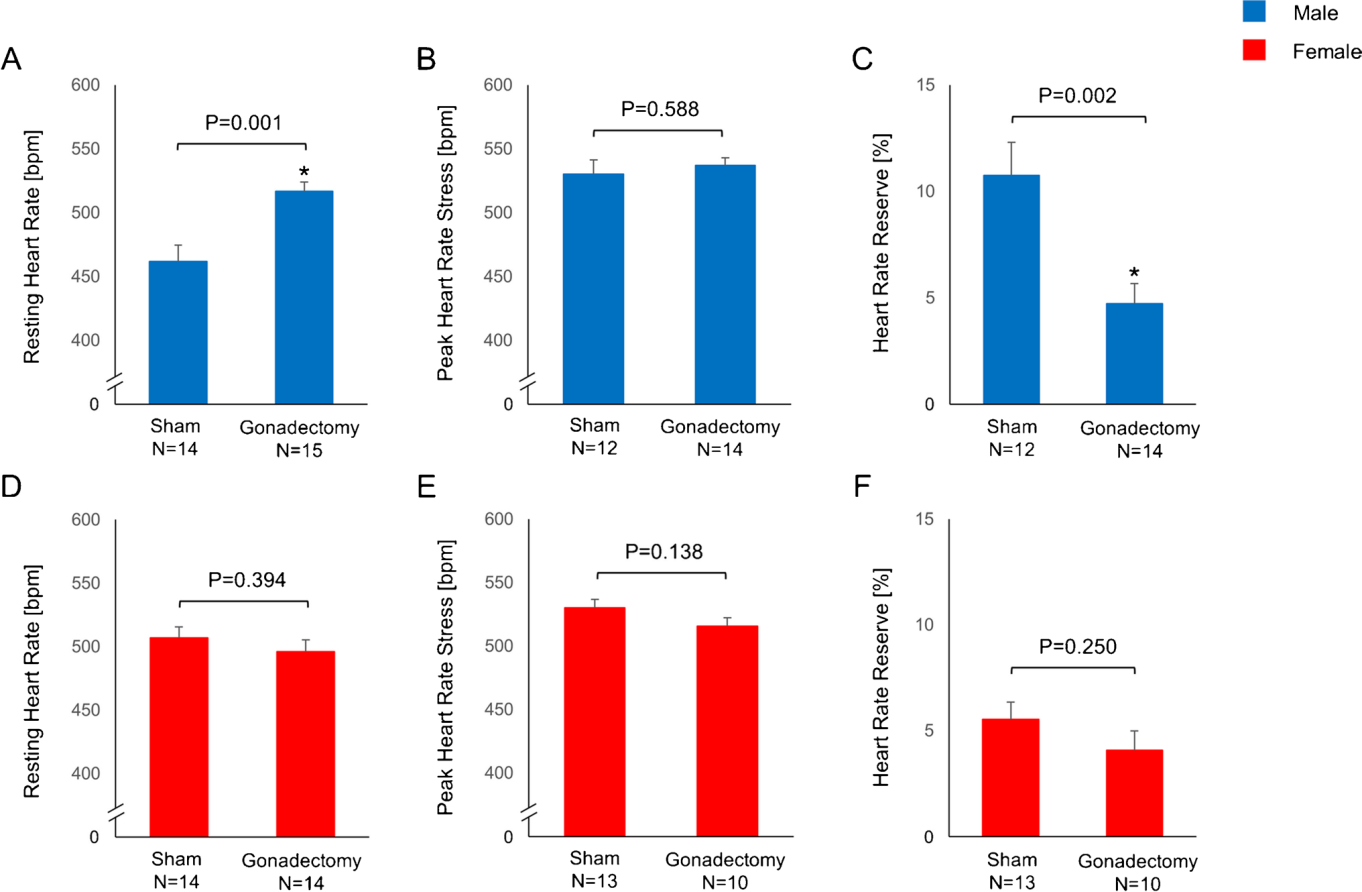
Heart rate and heart rate variability. (**A**) Baseline heart rate in males. (**B**) Stress heart rate in males. (**C**) Heart rate reserve in males. (**D**) Baseline heart rate in females. (**E**) Stress heart rate in females. (**F**) Heart rate reserve in females

Baseline heart rate was significantly higher in gonadectomized vs. sham males (517 ± 7 vs. 462 ± 13 bpm, *p* = 0.001, Fig. 3A), while this effect of gonadectomy on heart rate was not observed following administration of regadenoson (sham vs. Gx, 530 ± 11 vs. 537 ± 6 bpm, *p* = 0.588, Fig. 3B). Heart rate reserve during pharmacological stress was significantly higher in sham vs. gonadectomized males (11 ± 2 vs. 5 ± 1%, *p* = 0.002, Fig. 3C). Conversely, gonadectomy did not have an effect on heart rate reserve in female animals, as evidenced by the comparable heart rate at baseline and following vasodilator stress (Fig. 3D–F).

### Effect of gonadectomy on myocardial function

**Fig. 4:**
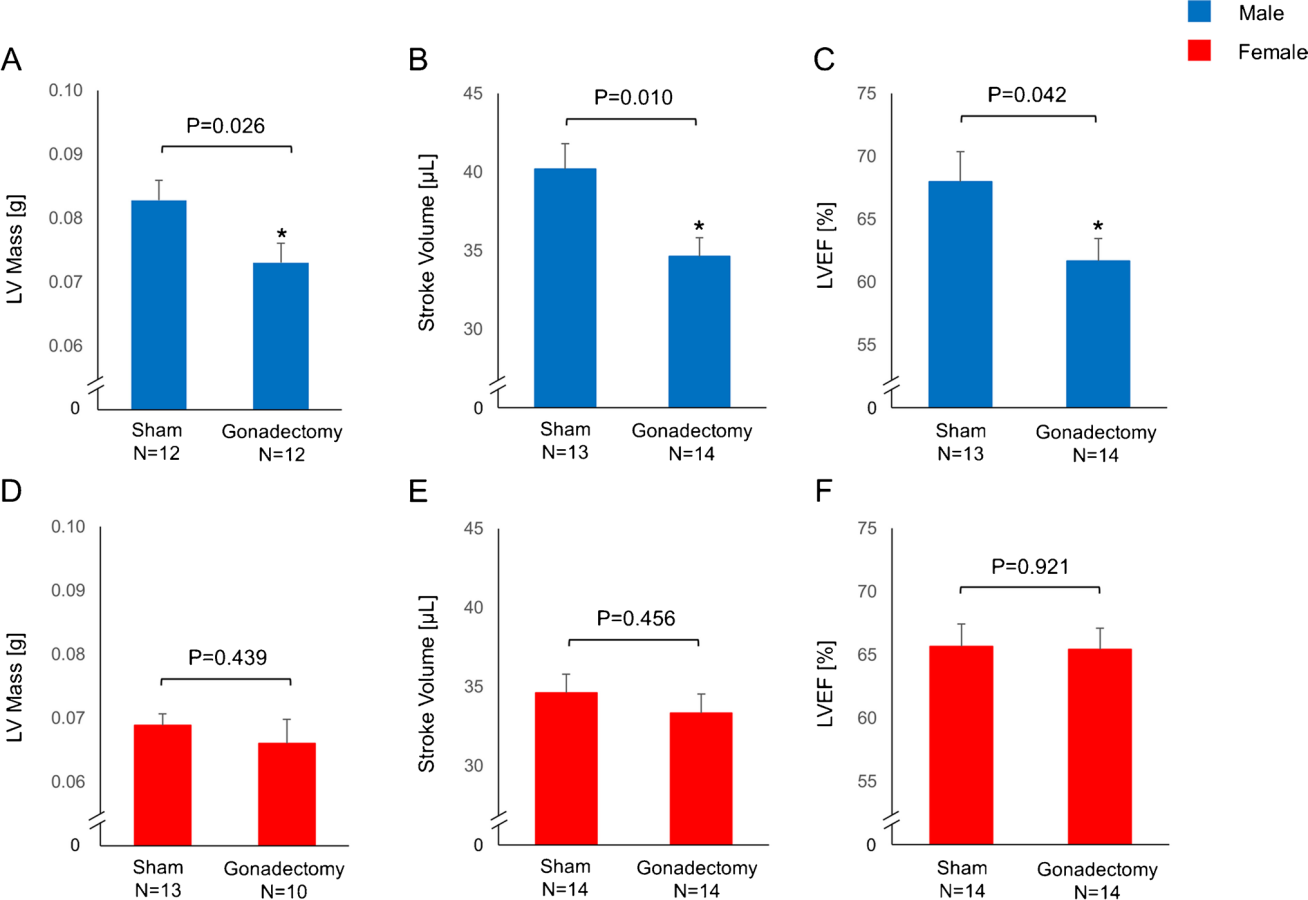
Cardiac magnetic resonance (CMR) imaging-derived left ventricular (LV) mass and function. (**A**) LV mass in males. (**B**) Stroke volume in males. (**C**) Left ventricular ejection fraction (LVEF) in males. (**D**) LV mass in females. (**E**) Stroke volume in females. (**F**) LVEF in females

No significant differences in body weight were observed between gonadectomized and sham animals (Supplemental Fig. 2A and D) in both males and females. Orchiectomy led to a significant reduction of LV mass in males (sham vs. Gx, 0.083 ± 0.003 vs. 0.073 ± 0.003 g, p = 0.026, Fig. 4A), while this effect was not observed following oophorectomy in females (0.069 ± 0.002 vs. 0.066 ± 0.004 g, p = 0.439, Fig. 4D). Further, orchiectomy resulted in a reduction of CMR-derived left ventricular stroke volume (sham vs. Gx, 40 ± 2 vs. 35 ± 1 μL, *p* = 0.010, Fig. 4B) and LVEF (sham vs. Gx, 68 ± 2 vs. 62 ± 2%, p = 0.042, Fig. 4C). The gonadectomy-induced decrease of LVEF in males was a consequence of slightly enhanced end-systolic volumes along with moderately decreased end-diastolic volumes in gonadectomized males (Supplemental Fig. 2B and C). In contrast, oophorectomy did not affect stroke volume (sham vs. Gx, 35 ± 1 vs. 33 ± 1 μL, *p* = 0.456, Fig. 4E) and LVEF (sham vs. Gx, 66 ± 2 vs. 65 ± 2 %, *p* = 0.921, Fig. 4F) in our study.

### Testosterone serum levels are associated with resting heart rate and myocardial perfusion

Low serum testosterone levels were associated with a blunted resting myocardial perfusion (*r* = 0.438, *p* = 0.020) and enhanced baseline heart rate in males (*r* = −0.526, p = 0.004, Table 1). Similarly, serum testosterone levels significantly correlated with CFR_App_ (*r* = −0.500, *p* = 0.007) and with heart rate reserve (*r* = 0.430, *p* = 0.032).

**Table 1:** Correlation of serum testosterone levels with key parameters of myocardial perfusion and heart rate variability in the male study population

Parameter	Resting myocardial perfusion	Stress myocardial perfusion	Apparent coronary flow reserve	Resting heart rate	Stress heart rate	Heart rate reserve
*r* (Spearman)	**0.438**	0.041	**−0.500**	**−0.526**	**−**0.154	**0.430**
P value	**0.020**	0.835	**0.007**	**0.004**	0.452	**0.032**
N	28	28	28	28	26	26

## Discussion

In the present experimental study, withdrawal of male, but not female, sex hormones increased CFR_App_, which was driven by a substantial reduction in resting myocardial perfusion. Accordingly, serum testosterone levels in male mice significantly correlated with resting myocardial perfusion and CFR_App_ on PET-MPI. Our results indicate that the reduced resting myocardial perfusion observed in testosterone-deprived males might be the consequence of a blunted HRR in these mice, which typically reflects an impaired cardiac autonomic function (e.g., reduced cardiac sympathetic outflow). Along this line, the reduction in myocardial perfusion was linked to a reduced cardiac function on CMR, as evidenced by the reduced stroke volumes and LVEF in testosterone-deprived males. The concept that reduced baseline myocardial perfusion is a secondary effect of cardiac remodeling processes was further supported by the notion that orchiectomy, but not oophorectomy, prompted a significant reduction in LV mass, while no differences in body weight were observed between experimental groups.

Consistent with our findings, previous reports have linked high testosterone hormone levels to an increased coronary vasodilation. As such, testosterone was found to trigger vasodilation in an in vitro study in isolated rabbit coronary arteries [14]. Similarly, administration of testosterone resulted in dilated coronary arteries in anesthetized male and female dogs [15, 16]. Further, intracoronary administration of physiological androgen doses in male pigs induced vasodilation and increased coronary blood flow [17]. In contrast to previous reports, however, our study is the first to compare the effects of oophorectomy and orchiectomy on myocardial perfusion in vivo by PET-MPI using [^18^F]flurpiridaz, which to date provides the highest spatial resolution among contemporary PET-MPI probes. Further, by performing rest and stress PET-MPI, we demonstrate that the vasodilatory effect of testosterone translated into increased myocardial perfusion at resting conditions, but not following injection of the pharmacological stressor. While the pharmacological stressor prompted maximal vasodilation, independent of testosterone levels, our findings imply that testosterone may act as a key modulator of CFR_App_ by selectively increasing resting myocardial perfusion. Along this line, high testosterone exposure in sham males may have resulted in the observed sex difference in CFR_App_ values for sham animals as the latter was reversed by gonadectomy.

In agreement with previous reports, we found that low testosterone was associated with a reduction of LV mass [18, 19]. The latter may have contributed to the attenuated cardiac contractility in orchiectomized males as evidenced by their blunted stroke volumes. Of note, we observed that these changes were associated with an increase in baseline heart rate, which points toward a chronotropic adaptation to reduced cardiac contractility. Similarly, the attenuated baseline myocardial perfusion in orchiectomized males can be explained by a decreased blood supply as a consequence of a blunted HRR. Nonetheless, our study does not address the question whether lower resting myocardial perfusion in sham males results from a direct coronary vasodilatory effect of testosterone or whether it is the manifestation of distinct LV remodeling processes in sham vs. orchiectomized males.

To date, few exploratory clinical studies have assessed the role of female sex steroids on myocardial perfusion and CFR. In agreement with our findings, Campisi et al. found that short- and long-term administration of 17β-estradiol did not affect myocardial perfusion [20]. Similarly, Schwitter et al. demonstrated that 3-month oral administration of 17β-estradiol did not influence CFR, HR, or resting coronary sinus blood flow in postmenopausal women free of coronary artery disease (CAD) [21]. Moreover, administration of combined hormonal therapy (estrogen and progestin) to postmenopausal women at risk for CAD had no effect on CFR or myocardial perfusion [22]. Clinical studies assessing the association between testosterone and myocardial perfusion are limited. Consistent with our data, increased myocardial perfusion in areas supplied by unobstructed coronaries was detected by MRI following oral administration of testosterone for 8 weeks in 22 men with coronary artery disease [23]. These findings support the notion that testosterone may contribute to coronary vasodilation and an improved baseline perfusion of the healthy myocardium [24]. Nonetheless, other studies unveiled that testosterone administration increased the risk of major adverse cardiovascular events (MACE) [25–29]. Despite initial efforts to elucidate the role of sex hormones on myocardial perfusion, prospective clinical trials encompassing healthy individuals and CAD patients are currently lacking.

Previous studies in healthy individuals and patients with suspected CAD indicated that baseline myocardial blood flow is higher in women than in men [1, 6]. Conversely, we did not detect any sex differences in baseline myocardial perfusion in our mouse model. These seemingly divergent findings may be attributed to the age of the animals in our cohort. Indeed, while we used relatively young mice with high physiological sex hormone concentrations, the vast majority of reported clinical cardiovascular studies encompassed men at an advanced age with unknown testosterone levels. Given that testosterone substantially declines with age in men [30], it is conceivable that the attenuated testosterone levels in elderly men may have contributed to the lower MBF observed in these studies. Our findings further imply that caution is warranted with regard to the interpretation of PET-MPI findings in patients with testosterone levels outside of the physiological range and patients receiving androgen deprivation therapy. Notwithstanding the reported sex differences in baseline and hyperemic MBF, Murthy et al. found similar CFR values in men and women with suspected CAD [1]. The average age in the latter study was 61.2 years for men and 62.3 years for women, suggesting that these individuals had reduced sex hormone levels. Along this line, sex differences in CFR_App_ were only observed in sham animals and diminished following gonadectomy in our study. While a CFR < 2.0 is considered abnormal in humans according to current ECS guidelines [31] and is a strong predictor of major adverse cardiac events in both sexes [1], there are no such reference values for rodents. In fact, reported CFR values in healthy rodents can range from 1.4 to 3.2 [32–36]. The latter variability can be, at least in part, attributed to the distinct imaging modalities employed for small-animal MPI. Notably, although PET-MPI is the reference standard for CFR measurements in humans, studies on CFR measurements with rest/stress PET-MPI in rodents are scarce, potentially owing to technical challenges that result from the short half-life of conventional PET-MPI probes including [^13^N]ammonia and [^15^O]water. We anticipate that the availability of [^18^F]flurpiridaz, which allows PET-MPI in research facilities without an on-site cyclotron, will lead to an increased use of PET-MPI in rodents, thereby providing a valuable tool for CFR quantification by small animal imaging in future studies. Other factors that may directly or indirectly affect the measurement of CFR include the underlying animal strain, sex, and age. Indeed, our findings imply that sex hormones significantly affect the measurement of CFR. Similarly, others have observed age-dependent changes of myocardial perfusion and CFR in mice [36]. These findings underscore the challenge of defining CFR reference values in rodents.

There are limitations to this study that should be noted. First, the findings obtained from this study are based on rodent data and may not be directly extrapolated to humans. However, previous reports have shown that testosterone-mediated regulation of autonomic function seems to be largely conserved across species [37–39]. Second, although high concentrations of isoflurane may exert coronary vasodilator properties [40], studies have shown that mean systolic blood pressure and heart rate remain stable at isoflurane concentration up to 2% in mice [41, 42]. Accordingly, to minimize the vasodilating effect of isoflurane, concentrations were kept below 2% throughout all experiments. Third, perfusion changes in other organs may have affected the systemic [^18^F]flurpiridaz availability, and thus tracer delivery to the myocardium. Indeed, regadenoson-induced changes in abdominal arterial flow may have resulted in an overall underestimation of the CFR_App_ values in our study. In the absence of an arterial input function, the latter cannot be excluded. Nonetheless, [^18^F]flurpiridaz exhibits higher uptake values in the heart compared to most other major organs, which limits the influence of extracardiac perfusion variability [43]. Finally, while our study shows an association of testosterone serum levels with resting myocardial perfusion, CFR_App_ and baseline heart rate, establishing causality warrants further investigations into molecular pathways that translate testosterone effects into changes in coronary vasoregulation and cardiac adrenergic signaling.

In conclusion, our results indicate that sex differences in myocardial perfusion are primarily driven by testosterone. While orchiectomy and blunted serum testosterone levels were associated with a reduced baseline myocardial perfusion and an enhanced CFR_App_, respectively, these findings were not observed in females. To the best of our knowledge, this is the first study assessing the effect of orchiectomy and oophorectomy on myocardial perfusion and flow reserve using small animal PET. With regard to the translational relevance of the present findings, accounting for the variability in testosterone exposure may help to improve diagnostic accuracy of PET-derived CFR in specific patient populations and identify patient subpopulations that profit most from this diagnostic modality.

## Supplementary Information

ESM 1 (PNG 91 kb)

High Resolution Image (TIF 497 kb)

ESM 2 (PNG 213 kb)

High Resolution Image (TIF 639 kb)
