# Supplementary figures and images for: Role of sex hormones in modulating myocardial perfusion and coronary flow reserve
Source: Eur J Nucl Med Mol Imaging. 2022 Jan 13;49(7):2209–18. doi: 10.1007/s00259-022-05675-2 (PMC9165260; doi:10.1007/s00259-022-05675-2)

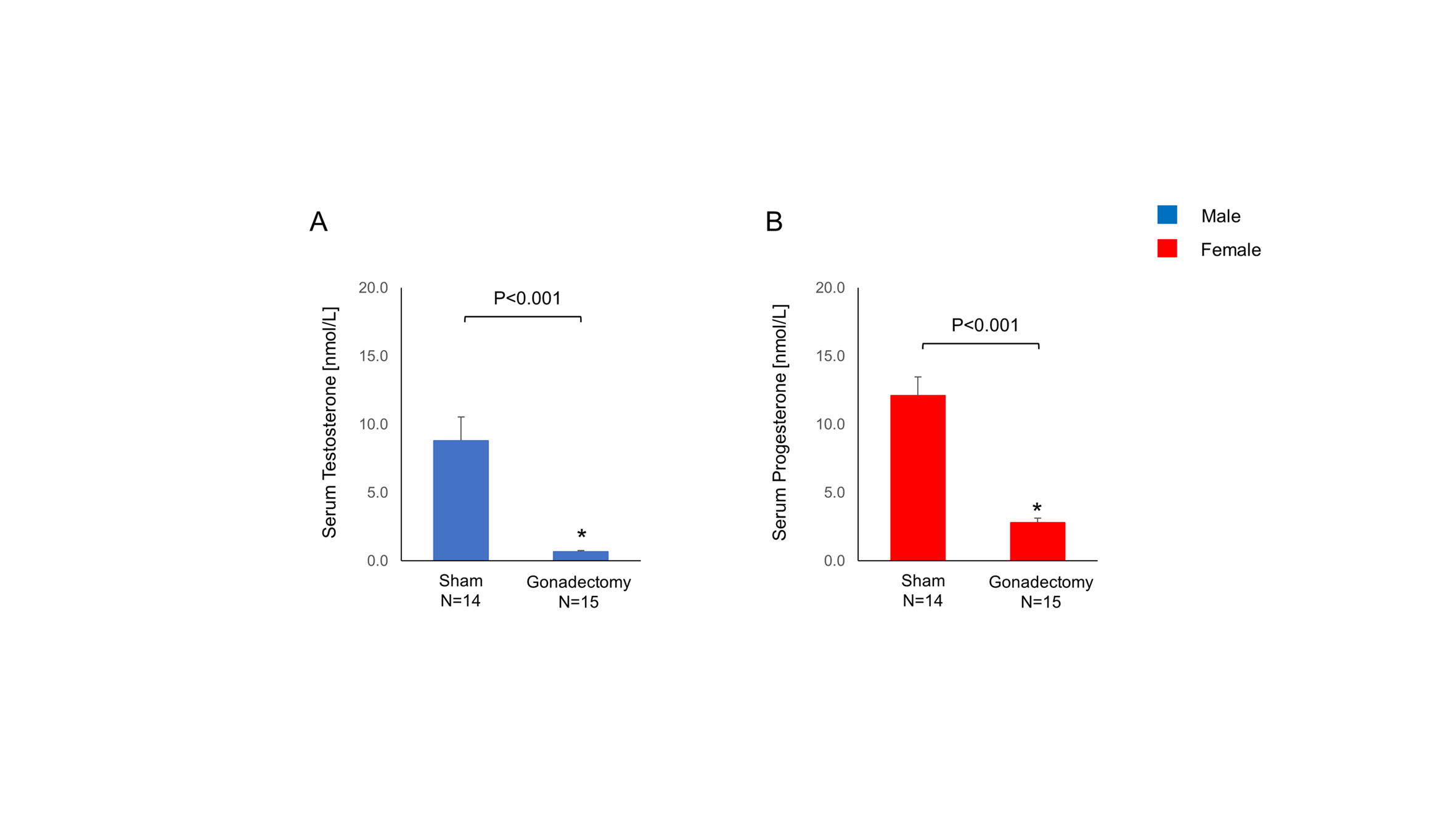

Supplement: Supplementary file 1 — (PNG 91 kb) [file 259_2022_5675_Fig5_ESM.png]

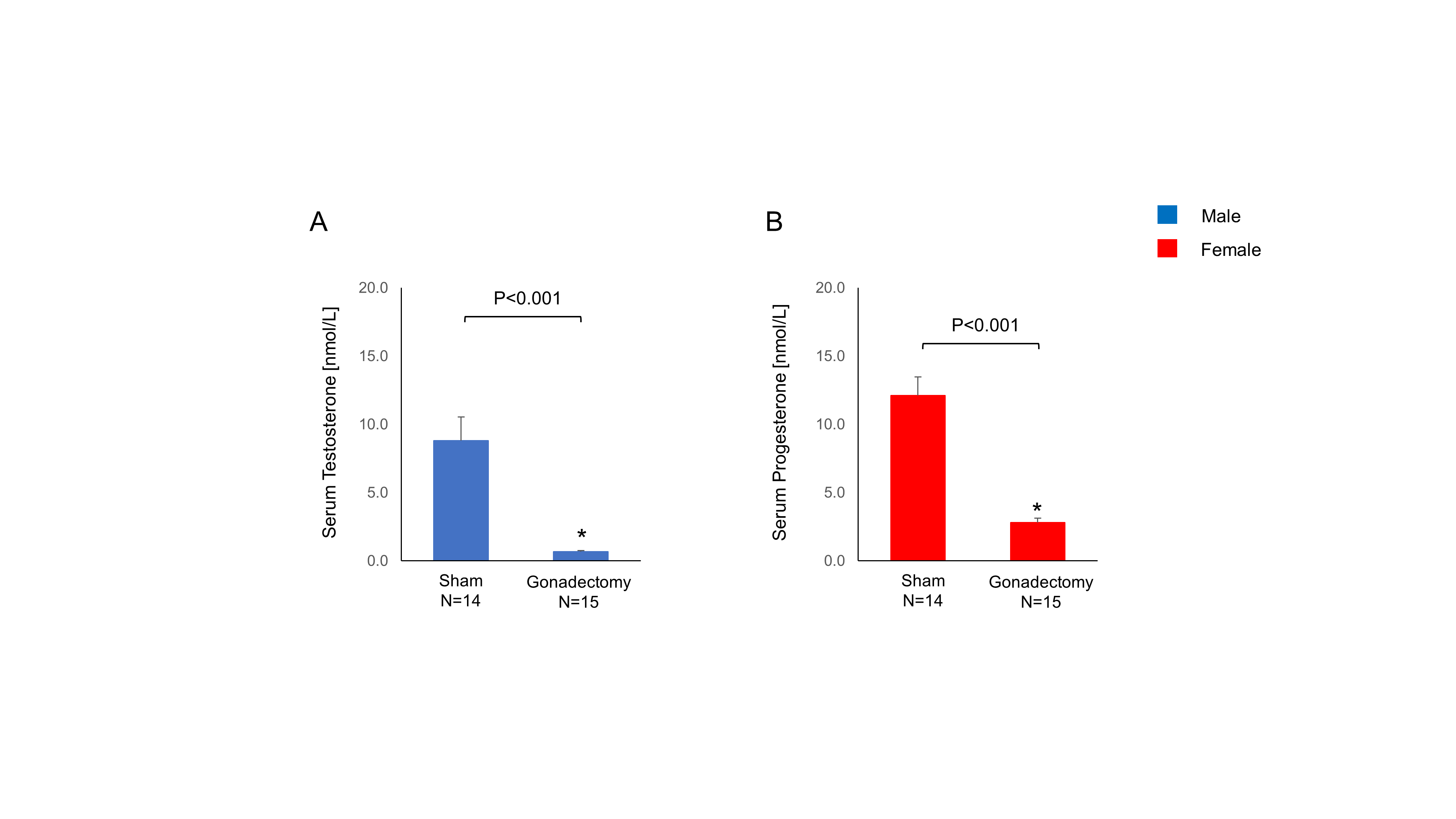

Supplement: Supplementary file 2 — High Resolution Image (TIF 497 kb) [file 259_2022_5675_MOESM1_ESM.tif]

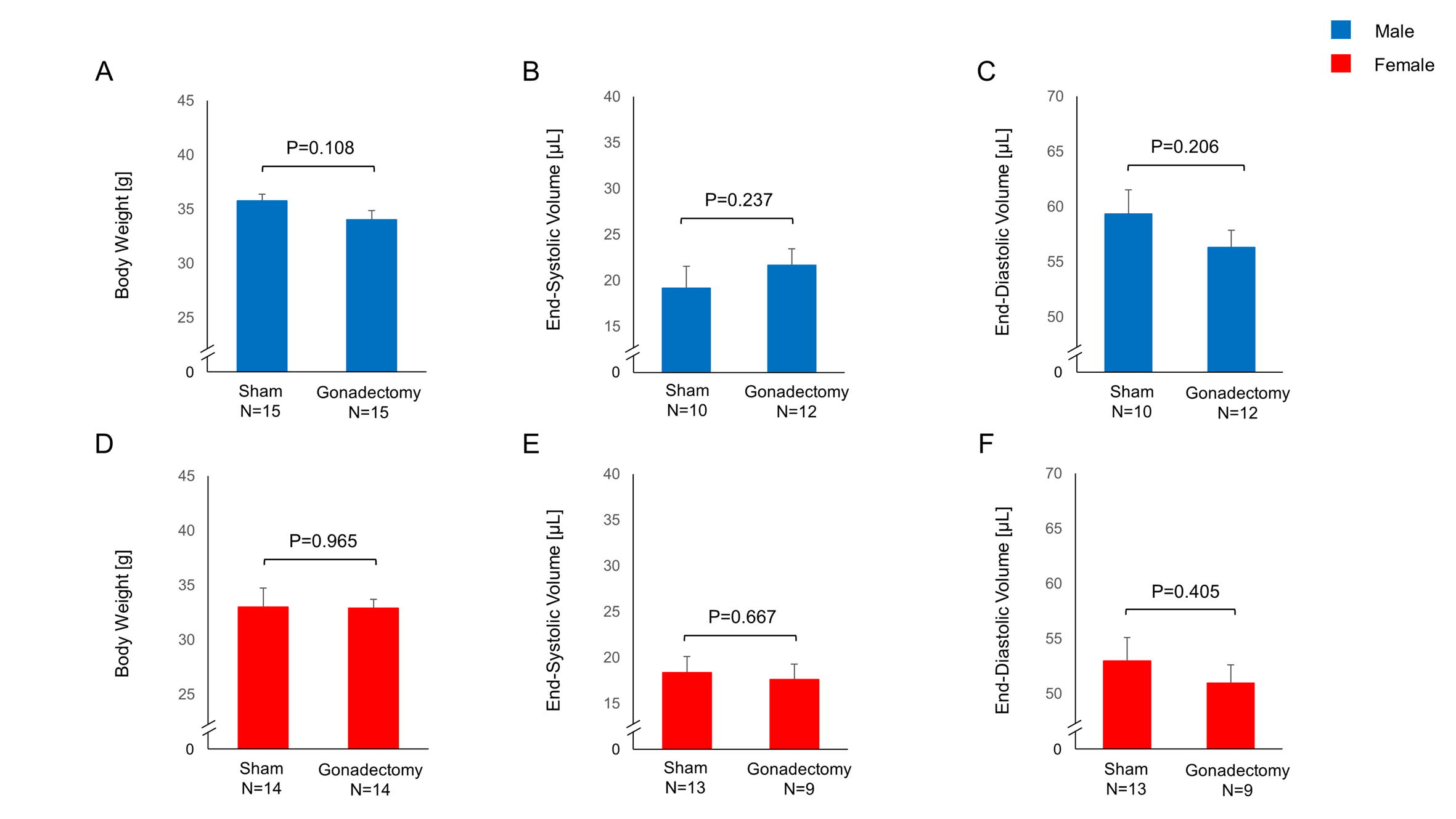

Supplement: Supplementary file 3 — (PNG 213 kb) [file 259_2022_5675_Fig6_ESM.png]

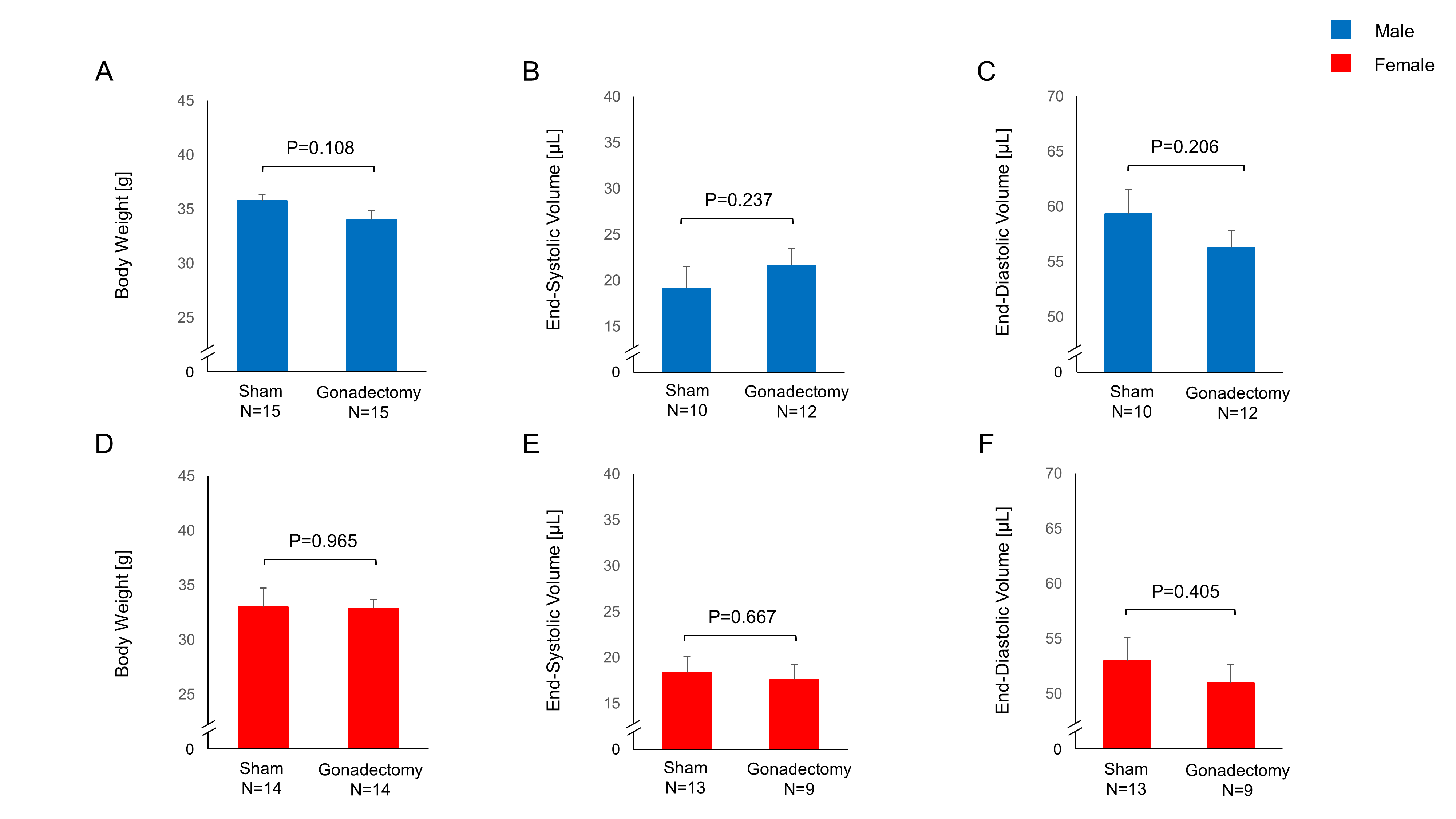

Supplement: Supplementary file 4 — High Resolution Image (TIF 639 kb) [file 259_2022_5675_MOESM2_ESM.tif]
